# Multimode hybrid gold-silicon nanoantennas for tailored nanoscale optical confinement

**DOI:** 10.1515/nanoph-2023-0105

**Published:** 2023-05-10

**Authors:** Cillian P. T. McPolin, Yago N. Vila, Alexey V. Krasavin, Jordi Llorca, Anatoly V. Zayats

**Affiliations:** Department of Physics and London Centre for Nanotechnology, King’s College London, Strand, London WC2R 2LS, UK; Universitat Politècnica de Catalunya, Escola Tècnica Superior d’Enginyeria de Telecomunicacions de Barcelona, Barcelona, Spain; Department of Chemical Engineering, Universitat Politècnica de Catalunya, EEBE, Barcelona, Spain

**Keywords:** cathodoluminescence, hybrid nanoantennas, Mie resonances, plasmonic nanostructures, silicon nanopillars

## Abstract

High-index dielectric nanoantennas, which provide an interplay between electric and magnetic modes, have been widely used as building blocks for a variety of devices and metasurfaces, both in linear and nonlinear regimes. Here, we investigate hybrid metal-semiconductor nanoantennas, consisting of a multimode silicon nanopillar core coated with a gold layer, that offer an enhanced degree of control over the mode selection and confinement, and emission of light on the nanoscale exploiting high-order electric and magnetic resonances. Cathodoluminescence spectra revealed a multitude of resonant modes supported by the nanoantennas due to hybridization of the Mie resonances of the core and the plasmonic resonances of the shell. Eigenmode analysis revealed the modes that exhibit enhanced field localization at the gold interface, together with high confinement within the nanopillar volume. Consequently, this architecture provides a flexible means of engineering nanoscale components with tailored optical modes and field confinement for a plethora of applications, including sensing, hot-electron photodetection and nanophotonics with cylindrical vector beams.

## 1 Introduction

The miniaturization of bulky optical elements to the nanoscale unlocks an abundance of disruptive photonic technologies [1], including novel metasurfaces [2] and metamaterials [3], whilst also enhancing existing technologies, such as sensors [4, 5], solar cells [6] and integrated photonics [7], [8], [9]. Optical nanoantennas are an essential building block in this regard, as they permit unprecedented control and confinement of light on the nanoscale and hence are particularly beneficial for sensing [10] and photodetection [11], lasing [12], optical trapping [13], and nonlinear optics [14]. A growing number of proposed nanoantenna designs are based on dielectric and semiconductor materials and support both electric and magnetic Mie-type resonances due to their nanoscale dimensions and high refractive indices. Silicon is widely used in this emerging technology due to its advantageous optical and electronic properties, together with the mature fabrication processes. Various designs have been studied in this context, including individual nanoantenna [14], waveguided [15] and metamaterial [16] geometries, with a cylindrical nanopillar shape being particularly suited for nanoantennas, both in terms of fabrication and efficient control of the supported optical modes.

Another well-developed approach for realizing high-performance nanoantennas relies on plasmonic excitations that result from the coupling of light to free-electron oscillations in metallic nanostructures [17]. Plasmonic nanoantennas have been used in numerous applications, including data storage [18], nanophotonic circuitry [8], neuromorphic computing [19], medicine [20] and nonlinear optics [21]. Importantly, metal-based schemes afford superior confinement compared to all-dielectric architectures and, therefore, hold a significant advantage with respect to field enhancement and sensing. In view of this, it is a natural next step to combine both approaches to realize nanoscale optical elements with low loss, large interaction volumes and enhanced near-fields. Multiple hybrid schemes in this vein have been previously proposed, including dielectric nanoparticles on metal substrates [22, 23], multilayered nanocavities [24, 25], Yagi-Uda nanoantennas [26], patch antennas [27, 28], bullseye structures [29], metaparticles [30], and composite hetero-nanoparticles [31, 32]. Such combined metal-dielectric architectures show substantial promise for nonlinear applications [33], single-photon sources [29], enhancing spontaneous emission [34, 35], integrated nanoscale lasing [36], sub-bandgap photodetection [37, 38], enhanced absorption [39], directional fluorescence [40], mode hybridization [41], and the generation of anapoles [42]. Importantly, further opportunities for development in this area include realizing hybrid schemes that provide tailored and tunable nanoscale optical confinement, further diversifying potential applications.

In this context, we have developed hybrid multimode nanoantennas consisting of silicon nanopillars supporting multiple resonant modes coated with a thin gold film, thereby concurrently enabling low losses, high optical confinement, and enhanced near-fields. High-refractive-index materials are particularly important for engineering the multipolar contributions in the optical response at the subwavelength scale, which makes them markedly advantageous for the realization of metamaterials and metasurfaces. High index structures also provide an opportunity for stronger field confinement at a metal/dielectric boundary. In order to elucidate the plethora of modes present in the nanopillars and their selection by the plasmonic coating, we employed cathodoluminescence (CL) imaging spectroscopy, which allows their optical properties to be mapped with nanoscale resolution. Electron beam excitation serves as a point-like broadband optical source, permitting the optical response to be mapped in tandem with conventional electron imaging [43], [44], [45]. This method was successfully applied for visualization and classification of the resonances of semiconductor AlGaAs nanopillars [46] and silicon nanocavities [47]. We show that the metal coating, which strongly affects the local density of optical states [48], provides an additional means of tuning the resonant response of the nanopillars, offering the opportunity for mode selection for the desired optical processes.

## 2 Results and discussion

**Figure 1:**
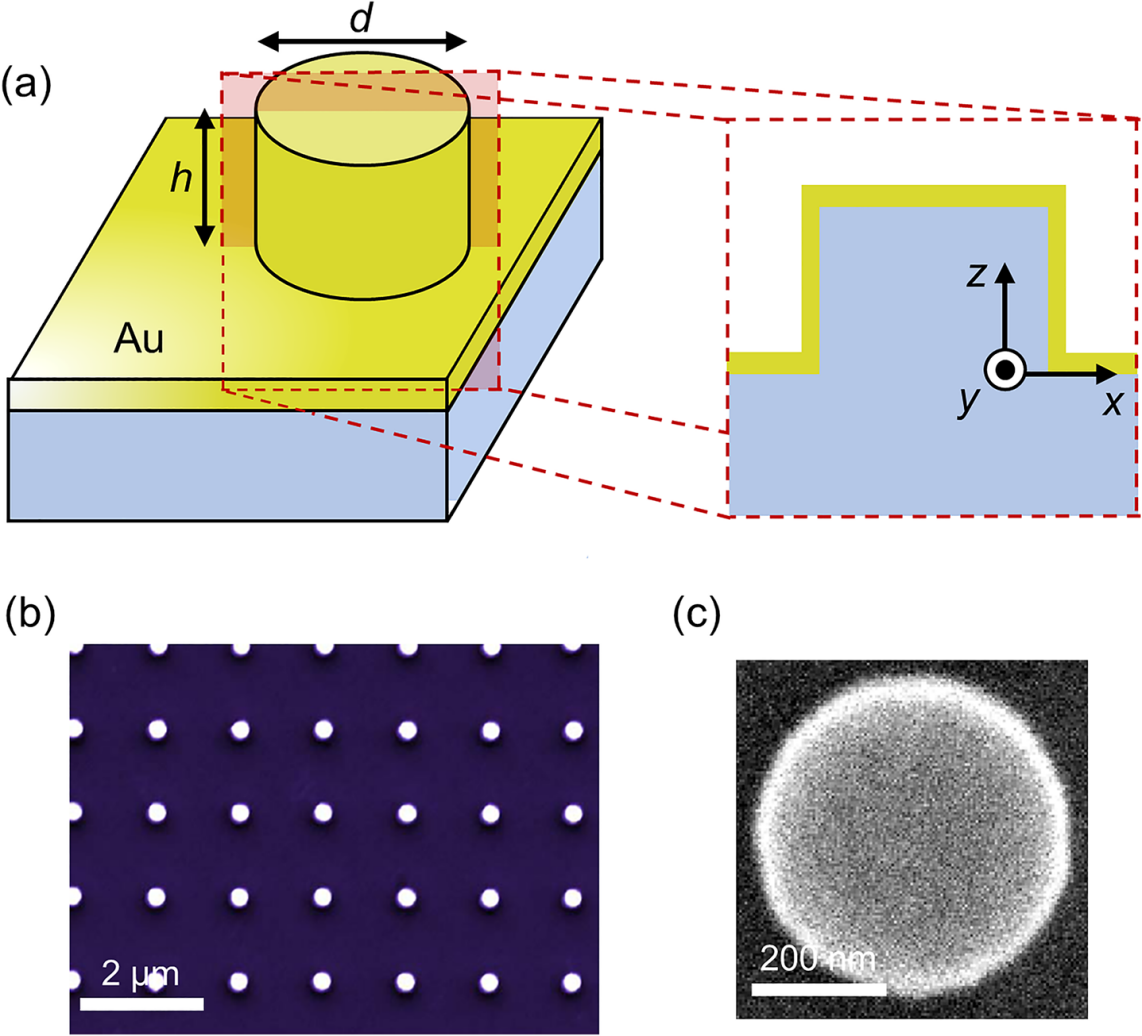
Hybrid gold-silicon nanoantennas. (a) Schematics of nanopillars with a diameter, *d*, and a height, *h*, patterned onto a silicon substrate and covered in a thin gold film. (b) and (c) SEM images (top views) of (b) a bare Si nanopillar array and (c) an individual Si nanopillar after coating with Au.

In order to reveal the nature of the optical response of the hybrid multimode nanopillars (Figure 1), we investigated the optical modes supported by both Au–Si and bare Si nanopillars of a wavelength size using CL emission and subsequently compared them to numerical simulations (for the details of fabrication, measurement and simulations, see Methods). CL imaging spectroscopy probes far-field radiation [49] and, therefore, the experimental spectra and maps correspond to the modes that are able to be efficiently excited by the e-beam and also radiate well into the far-field.

**Figure 2:**
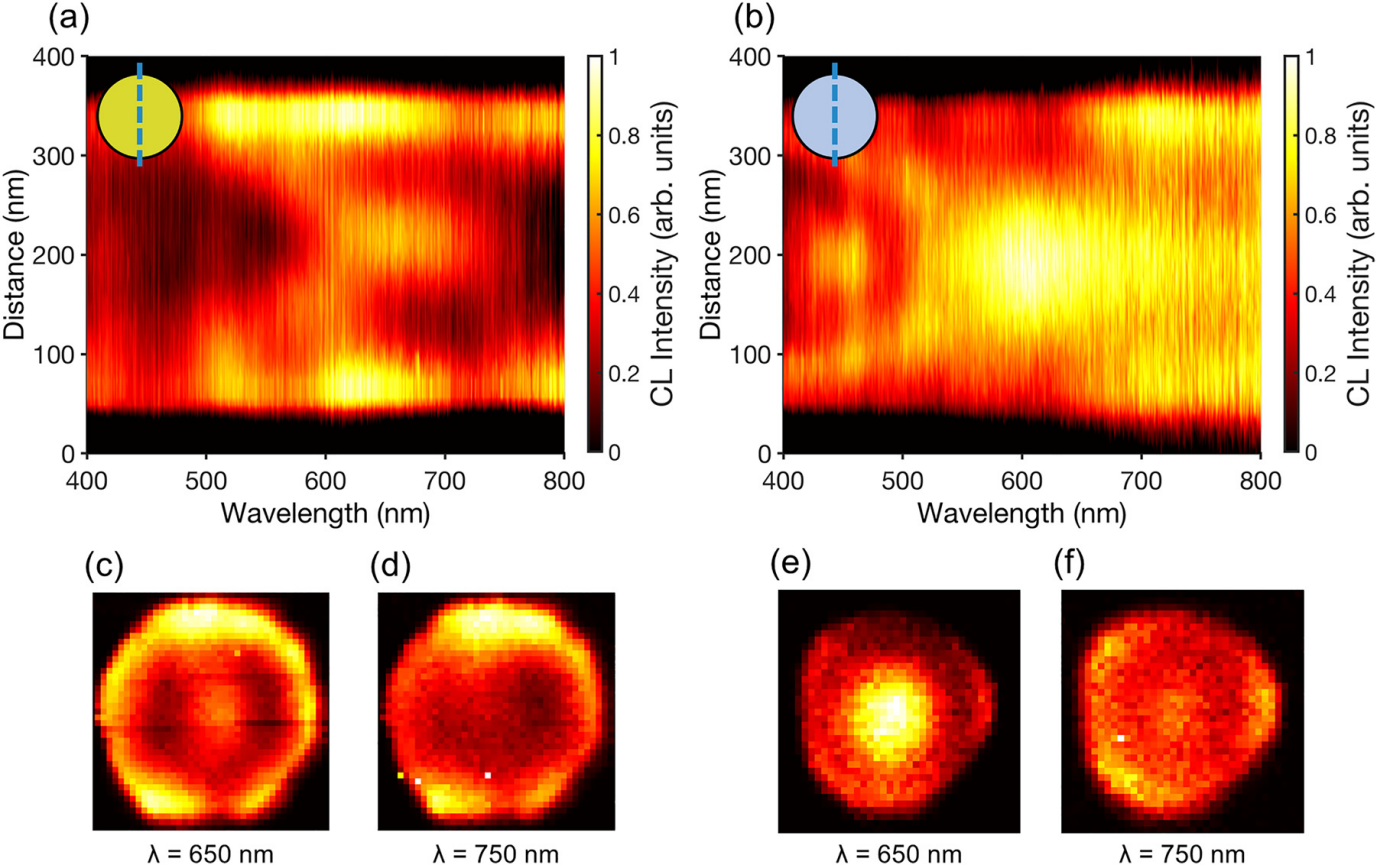
CL emission from (a), (c), (d) Au–Si and (b), (e), (f) Si nanopillars. (a) and (b) The dependence of the CL spectra on the e-beam position across the nanopillar (as shown in the inserts). (c)–(f) CL intensity maps at wavelengths of (c) and (e) 650 nm and (d) and (f) 750 nm. The bandwidth for each CL map is 30 nm. The CL intensity in each figure has been normalized to 1.

The influence of the metal coating is made apparent by comparing the CL emission from Au–Si and Si nanopillars (Figure 2). The CL spectra, measured at different positions of the excitation beam, illustrate the variation of the spectral response of the nanopillars as the e-beam is moved across the nanopillar facet. Considerably stronger CL emission is observed when the e-beam is positioned at the edges of the Au–Si nanopillar compared the center (Figure 2(a)). However, this trend is reversed in the case of the bare Si nanopillar (Figure 2(b)). This spatial variation in the spectra is also highlighted by the corresponding CL maps (Figure 2(c)–(f)), suggesting that different modes are dominant in the case of Si and Au/Si nanopillars. Efficient excitation of a particular mode takes place when its electric field and the localized electric field generated by the incident electron beam have a good overlap in terms of spatial and polarization distributions. For instance, in the simplest case of dipolar plasmonic modes supported by small metal nanoparticles, it is expected that out-of-plane modes are preferentially excited when the e-beam is placed in the center of a nanopillar, whereas in-plane modes are excited more efficiently in the case of the excitation at the edge [50].

**Figure 3:**
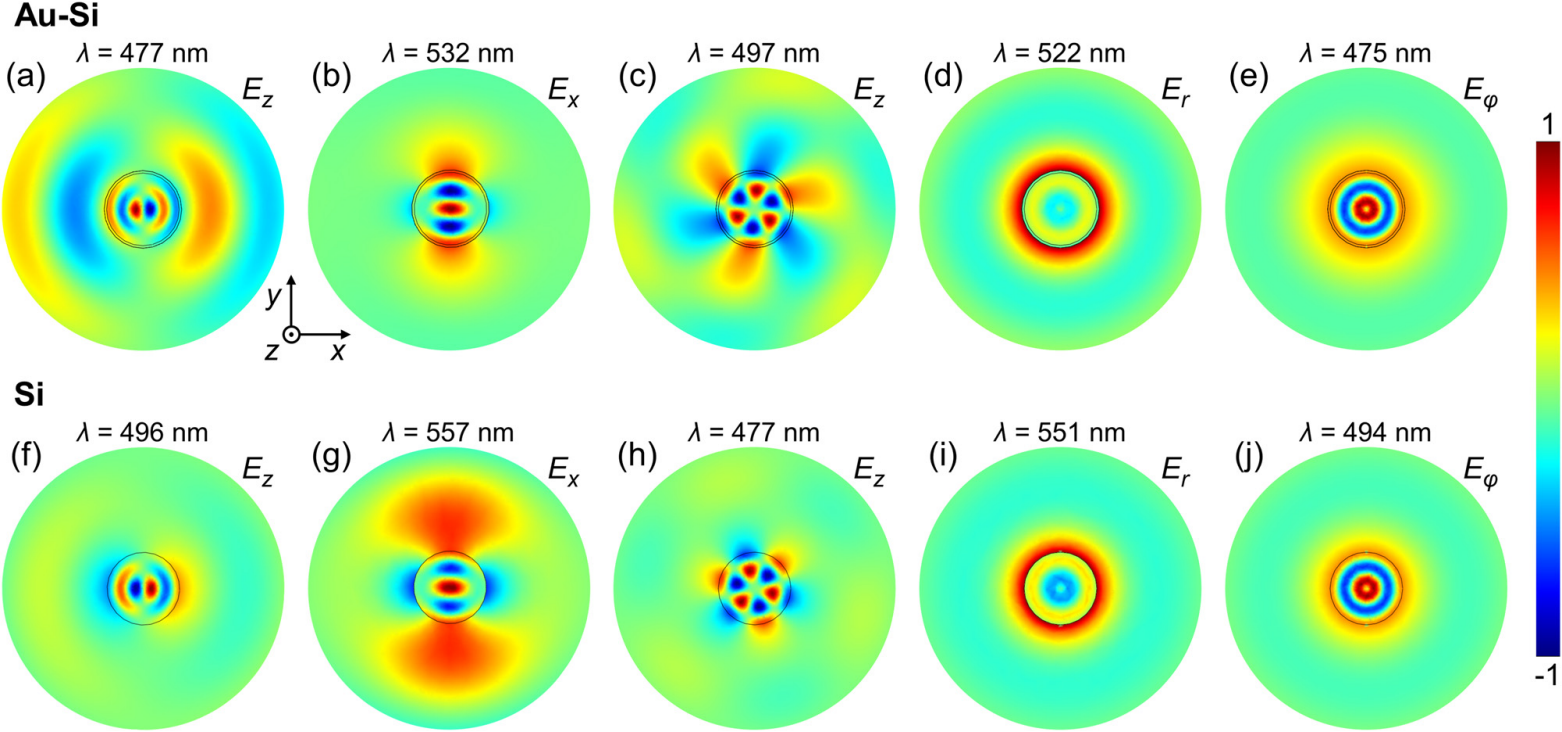
Numerically simulated electric field maps of various types of the eigenmodes supported by (a)–(e) Au–Si and (f)–(j) Si nanopillars. (a) and (f) Vertically (*z*-) polarized, (b) and (g) in-plane (*xy*-) polarized, (c) and (f) whispering gallery, (d) and (i) radially polarized and (e) and (j) azimuthally polarized modes. The cross-section vertical position is at the half-height of the Si nanopillar (*z* = 120 nm).

As the bare Si nanopillars do not support plasmonic modes, the optical emission can be solely attributed to the presence of Mie-type resonances. However, in the case of Au-coated Si pillar, in addition to localized modes, the incident e-beam will also generate surface plasmon polaritons (SPPs) that propagate on the nanopillar surface and gold surface between them (Figure 1). These SPP excitations may also contribute to the observed spectra after scattering at the nanopillar edges. Overall, the CL maps represent spectrally- and position-dependent excitation of numerous (many dozens) optical modes, supported due to the high refractive index of the nanopillar silicon core. To reveal their exact character numerical simulations of both Si and Si-Au nanostructures have been performed. The observed optical modes have a diverse nature, but can be classified into vertically (*z*-) polarized, in-plane (*xy*-) polarized, whispering gallery, radially polarized, and azimuthally polarized types (characteristic examples are presented in Figure 3). It is interesting to note that the structure exhibits a resonant behavior for cylindrical vector modes with radial and azimuthal polarization, which can be useful for complex vector beam manipulation [51]. Significantly, there is an enhanced field at both the Au–Air and Au–Si interfaces; the former would be especially beneficial for sensing, whilst the latter could lend itself to photodetection applications [38]. The optical response of the hybrid nanopillars is, therefore, highly dependent on the wavelength and position of excitation. This enables an opportunity to deliberately position emitters on the top of the nanopillars to induce a more plasmonic/Mie-type or hybrid response, depending on the position and emission wavelength. Furthermore, greater control over the confinement may also be afforded by coupling between adjacent nanopillars with a nanoscale gap [46]. We note, that the absolute comparison of CL intensities for Si and Au–Si nanopillars is unreliable, as in each case realignment of the setup is needed after the sample change, which may affects the collection efficiency to up to ∼30 % [52]. Therefore, only the relative excitation efficiencies of different modes in the bare Si and Au–Si nanopillars are compared.

**Figure 4:**
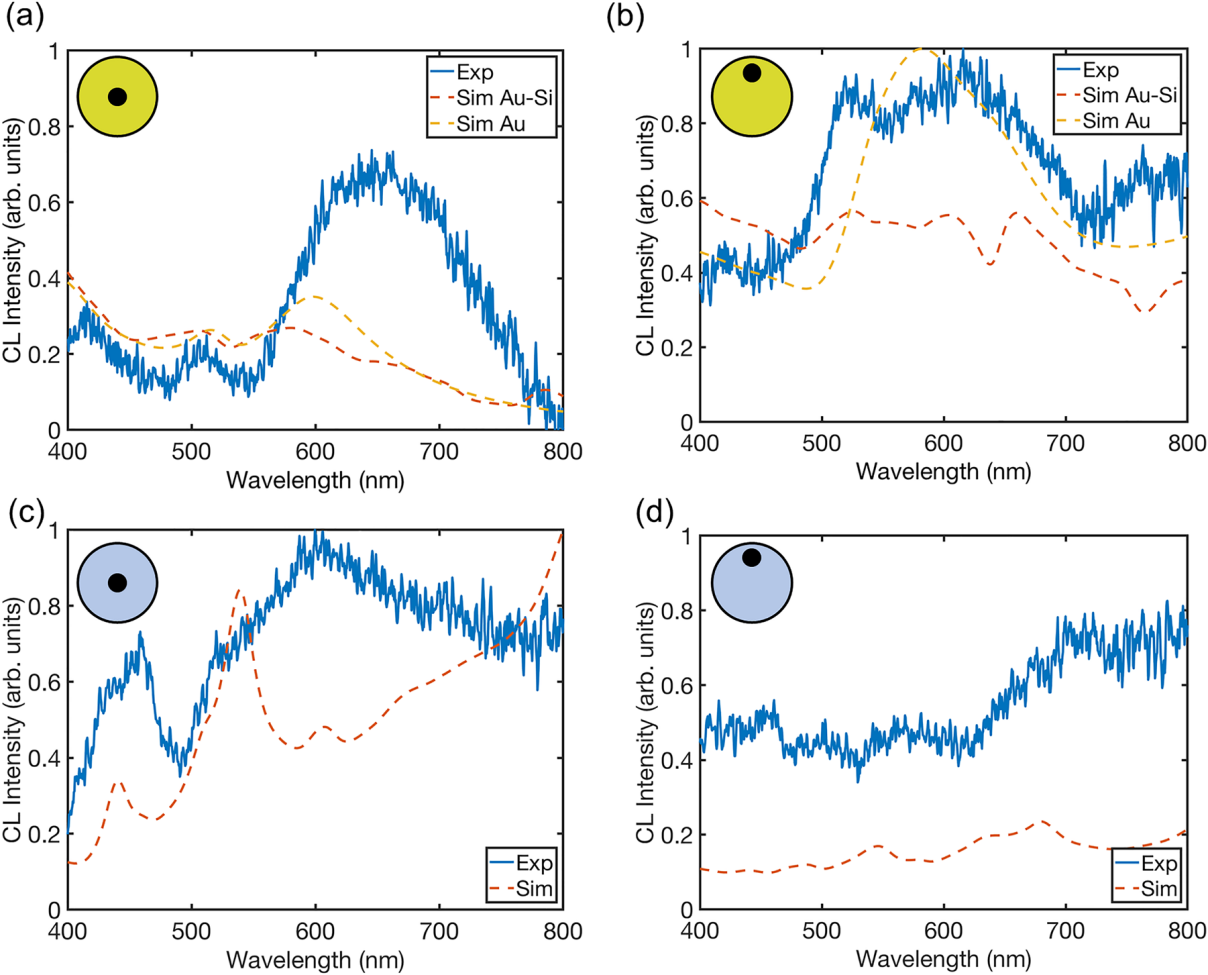
Experimental (Exp) and simulated (Sim) CL spectra for (a) and (b) Au–Si and (c) and (d) Si nanopillars. The e-beam excitation is positioned in the center of the nanopillars in (a) and (c) and at the edge in (b) and (d). Corresponding simulations for a solid Au nanopillar are also included for comparison in (a) and (b). The experimental and simulated Au–Si and Au spectra in (a) and (b) and (c) and (d) have been normalized to 1 for all the graphs in each pair.

Comparing the experimental and simulated spectra of the nanopillars at the different positions of the e-beam excitation (Figure 4), good agreement in terms of peak positions is observed (the spectra simulated for a solid Au nanopillar are also presented for reference). Broader than expected experimental peaks are generally observed, which may be due to a departure from circular symmetry of the nanopillar experimental geometry. In the latter case, the degeneracy of the modes is lifted, giving rise to multiple overlapping peaks. The spectral shift between the experimental and simulated results can also be partly accounted for by variations in the nanopillar diameter. In the case of Au-Si structures, it can be due to the variations in the gold layer thickness from the estimated value and gold permittivity (which depends on the particular deposition method) from the tabulated data. For these structures, it can be also related to the underestimation of the role of incoherent CL sources in Si in numerical modelling. In Refs. [53, 54], a good agreement with the experimental data was obtained for plasmonic structures considering the coherent CL source to be dominant, while Ref. [52] suggests that the incoherent part can be substantial, at least comparing incoherent CL from Si with the coherent counterpart from bulk Al. We have chosen the modelling approach as in Refs. [53, 54] due to similarity of metal/semiconductor systems studied, but the incoherent CL, which is not captured in the simulations, may still be present in the experiment and its contribution maybe different at different wavelengths. For the gold nanopillars, when the e-beam is located in the center (Figure 4(a)), there is a strong resemblance between the Au–Si and Au spectra, indicating that the plasmonic response dominates. However, when the nanopillar is excited at the edge (Figure 4(b)), the Au–Si nanopillars exhibits additional spectral features compared to the solid Au, marking the influence of the silicon core on their response.

**Figure 5:**
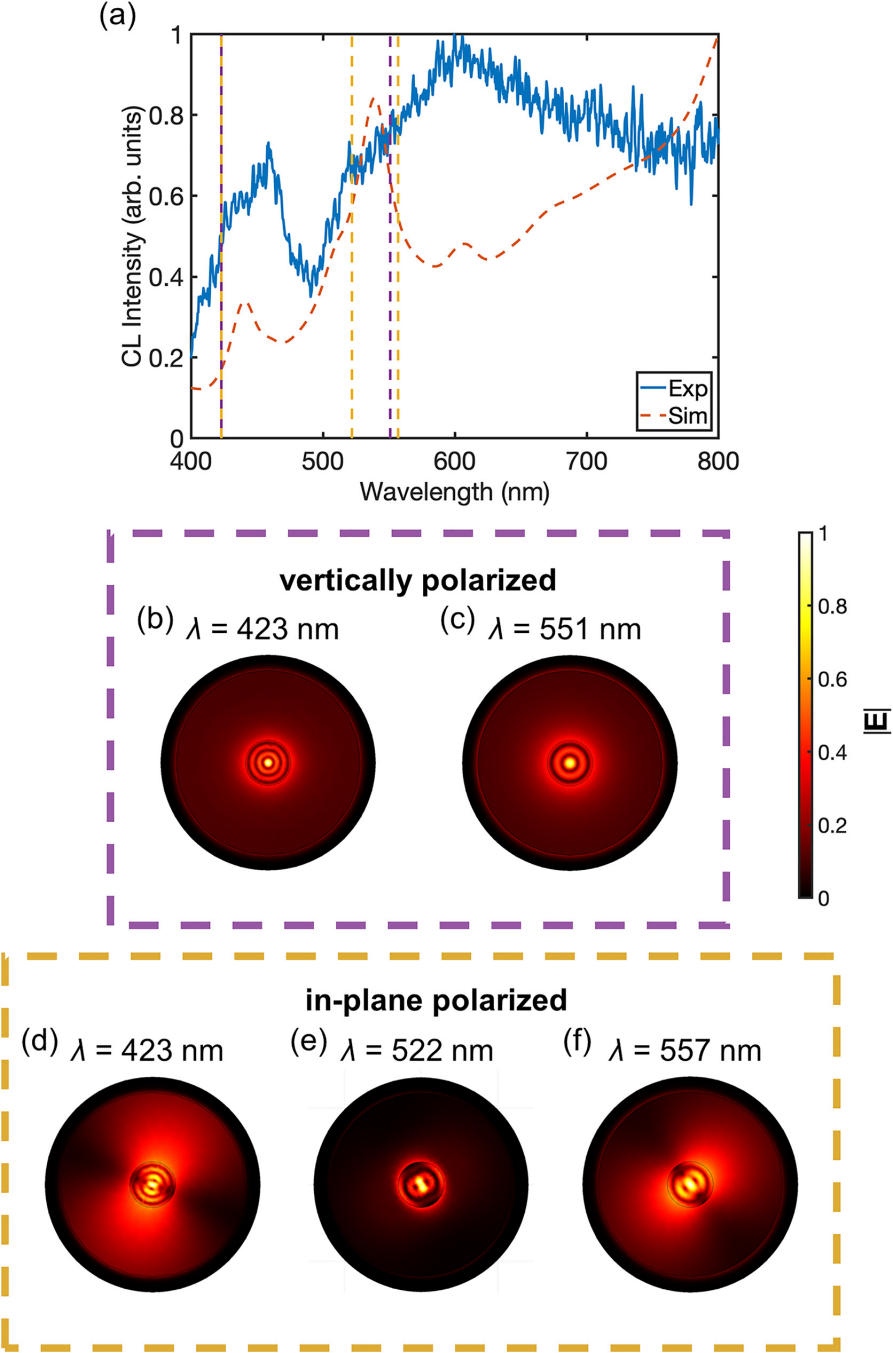
Eigenmode analysis of a CL spectrum of a Si nanopillar with e-beam excitation in the center. (a) Experimental (Exp) and simulated (Sim) CL spectra for a Si nanopillar with a diameter of 300 nm. (b)–(e) Eigenmode |**E**| field maps in the transverse plane at the wavelengths marked in (a): modes in (b) and (c) are vertically (*z*-) polarized, while in (d)–(f) are in-plane (*xy*-) polarized. The cross-section vertical position is at the half-height of the Si nanopillar (*z* = 120 nm).

As confirmed by an eigenmode numerical analysis, a significant number of modes are supported by the bare silicon nanopillars with a diameter comparable to the wavelength, which spectrally overlap, giving rise to broad peaks. For instance, for *d* = 300 nm and *h* = 240 nm, there are dozens of modes supported by the pillar in the studied wavelength range, though only 5 were found to be efficiently excited when the electron-beam is located in the center due to the symmetry considerations (Figure 5). Plasmonic modes, arising due to the presence of the gold film, also contribute to the spectra, leading to a more complex response and damping or enhancing specific modes, allowing modification and the selection of the mode spectrum from the available spectrum of the modes of a bare Si pillar. Moreover, as the CL emission collected here is predominately coherent (i.e. there is a coherent phase link between the emitted CL and the electric field associated with the exciting e-beam), interference may occur between various modes, leading to the partial cancellation of out-of-phase modes, depending on the emission direction, and, therefore, their suppression or shift in the collected spectra. It is also important to note that far-field emission from the modes confined within the pillar volume will be attenuated due to absorption in the gold film and, thus, may not be clearly visible in the collected CL emission. Hence, the far-field response of the Au–Si nanopillar can be tuned from that resembling a plasmonic response to a more hybrid response by altering the position of the exciting e-beam as in this work or selectively placing other point-like emitters at a prescribed locations.

## 3 Conclusions

We investigated the interplay between the modes of the multimode dielectric nanoantenna covered with a plasmonic layer. In contrast to previous works on dielectric nanoantennas, which were focused on the nanontennas in the spectral range of electric and magnetic dipolar resonances, the system studied here is truly multimode and supports a variety of confined modes of different polarisations including cylindrical vector modes. We have successfully developed and characterized a multimode hybrid metal-dielectric platform for nanophotonic elements that provides greater control of light on the nanoscale compared to purely plasmonic or Mie-type structures and allows to select the required modes from the available mode spectrum. The developed system offers interesting opportunities for tailoring optical properties of nanoscale elements for a wide array of applications where the field confinement at the specific locations at plasmonic/semiconductor interfaces is needed at the specific wavelengths and with the specific polarisation distributions. The enhanced near-field afforded by the gold layer enables their use in sensing, whilst the large interaction volumes provided by the Si core and field enhancements due to plasmonic coating is additionally beneficial for nonlinear applications. Interference between the various nanopillar modes may also be harnessed to potentially provide highly directional emission. Such multimode hybrid antennas may serve as a versatile platform for future tuneable nanoscale optical components, offering low-loss and high field confinement across a wide range of optical technologies, including sensing, photodetection, photochemistry, nonlinear optics, complex vortex beams manipulation, and other integrated photonic devices.

## 4 Methods

### 4.1 Fabrication

Bare Si nanopillars with a diameter of 300 nm and a height of 240 nm were patterned in the square arrays with a periodicity of 1500 nm using electron-beam lithography onto a Si wafer. A native oxide layer of a Si wafer was removed before coating with a negative tone resist. The structures were then patterned by electron beam with 100 keV energy. Dry etch was used to transfer the patterns onto Si with the required height. The fabricated structures were stored in air and a naturally formed native oxide layer was not removed for the measurements or before Au deposition. As the oxidation of silicon has a self-limiting character and the thickness of the native oxide layer is only ∼2 nm, such thickness is too small to noticeably affect the spectral positions and widths of the studied modes and, therefore, was not considered in the simulations. An Au layer subsequently deposited via magnetron sputtering (35 nm thick on the horizontal surfaces and 10 nm thick on the nanopillar sides). A schematic and an SEM image of the hybrid nanopillars are presented in Figure 1(a) and (b). The periodicity of the studied nanopillar arrays (1500 nm) allows to minimize the interaction between individual nanopillars and the plasmonic crystal effects resulting from the SPP band gap formation [55] are also absent in the studied visible spectral range, but maybe important in the near-infrared spectral range, where the SPP propagation length on Au is larger.

### 4.2 Cathodoluminescence measurements

Measurements of the CL emission were carried out using a commercial system (SPARC, Delmic). A focused 30 keV electron beam was scanned across the samples, resulting in light emission from the nanopillars which was collected by a parabolic mirror and directed into a spectrometer. A CL spectrum was collected for each beam position, producing a hyperspectral image for each nanopillar. The background CL emission measured from the nonpatterned part of the substrate was subtracted from each measurement, allowing the emission from individual nanostructures to be mapped. We note that this is impossible to achieve with conventional optical characterization, where a large number of pillars would be illuminated with a collimated beam. This would result in the spectra dominated by the periodic lattice effects, related to the diffraction. Similarly, strongly focused optical illumination, while addressing a single pillar, results in strongly scattered light which will interact with other pillars in the array, still resulting in the pronounced lattice effects.

### 4.3 Numerical simulations

Numerical simulations of the CL signal from the Si, Au–Si and Au nanostructures, and their eigenmodes, were performed using a finite element method (COMSOL Multiphysics software). The calculated CL signal consists of coherent and incoherent parts [45], summed with certain frequency-dependent coefficients which were found by matching the numerical results to the experimentally measured CL from a flat Si surface [52]. The incoherent signal was calculated using a line of randomly-oriented dipoles along the beam path in Si, presenting excitation and recombination of e-beam generated electron–hole pairs. Coherent CL signal was calculated using two dipoles positioned at the places where the e-beam hits the air–Au and Au–Si interfaces, corresponding to the collapsing dipoles produced by the electrons together with their images and having a phase distribution defined by a speed of the electrons. In the case of Au–Si (35 nm top and 10 nm side Au thicknesses) and Au nanostructures, the coherent CL component was considered to be dominant [53, 54]. For both coherent and incoherent components, the CL signal was calculated by integrating the emitted power flow over a hemisphere surrounding the nanostructure on the air side. The eigenmodes of the structure were found using a standard eigenmode solver. In either simulation mode were surrounded by perfectly matched layers to ensure the absence of back reflection.
